# Nest site preference depends on the relative density of conspecifics and heterospecifics in wild birds

**DOI:** 10.1186/s12983-017-0246-5

**Published:** 2017-12-20

**Authors:** Jelmer M. Samplonius, Iris M. Kromhout Van Der Meer, Christiaan Both

**Affiliations:** Conservation Ecology Group (CONSECO), Groningen Institute for Evolutionary Life Sciences (GELIFES), University of Groningen, PO Box 11103, 9700CC Groningen, the Netherlands

**Keywords:** Birds, Cultural evolution, *Ficedula hypoleuca*, Habitat selection, Heterospecific attraction, Interspecific competition, Paridae, Passerines, Public information, Social learning

## Abstract

**Background:**

Social learning allows animals to eavesdrop on ecologically relevant knowledge of competitors in their environment. This is especially important when selecting a habitat if individuals have relatively little personal information on habitat quality. It is known that birds can use both conspecific and heterospecific information for social learning, but little is known about the relative importance of each information type. If provided with the choice between them, we expected that animals should copy the behaviour of conspecifics, as these confer the best information for that species. We tested this hypothesis in the field for Pied Flycatchers *Ficedula hypoleuca* arriving at their breeding grounds to select a nest box for breeding. We assigned arbitrary symbols to nest boxes of breeding pied flycatchers (conspecifics) and blue and great tits, *Cyanistes caeruleus* and *Parus major* (heterospecifics), in 2014 and 2016 in two areas with different densities of tits and flycatchers. After ca 50% of flycatchers had returned and a flycatcher symbol was assigned to their nest box, we gave the later arriving flycatchers the choice between empty nest boxes with either a conspecific (flycatcher) or a heterospecific (tit) symbol.

**Results:**

As expected, Pied Flycatchers copied the perceived nest box choice of conspecifics, but only in areas that were dominated by flycatchers. Against our initial expectation, flycatchers copied the perceived choice of heterospecifics in the area heavily dominated by tits, even though conspecific minority information was present.

**Conclusions:**

Our results confirm that the relative density of conspecifics and heterospecifics modulates the propensity to copy or reject novel behavioural traits. By contrasting conspecific and heterospecific ecology in the same study design we were able to draw more general conclusions about the role of fluctuating densities on social information use.

## Background

Finding a suitable breeding location is a demanding process for cavity nesting birds, as components affecting suitability fluctuate spatially and temporally [1]. Proximate factors affecting habitat selection may include structural properties, nesting suitability, foraging opportunities, or the presence of other individuals. Habitat information may be acquired by personally sampling the habitat, or by eavesdropping on the decisions of others, coined social information use [2]. Theoretically these strategies can be considered a producer scrounger game [3], in which some individuals gather personal information about habitat characteristics (producers), and others eavesdrop on information gathered by their competitors (scroungers). Producers may also be referred to as samplers, and scroungers as cue-users [4], reflecting the basic two strategies animals can follow to plastically respond to fluctuating environments. There may be several trade-offs modulating the propensity to use personal or social information, and it is generally thought that social learning should be preferred when individual learning is more costly than eavesdropping [5]. For example, it is expected that scrounging is under frequency dependent selection, as information becomes completely meaningless when every individual scrounges [3]. Time pressure may also modify the propensity to prefer scrounging over sampling: later arriving flycatcher individuals with little time between arrival and breeding were more likely to use social cues of tits than earlier individuals [6, 7].

Social information can be gathered from conspecific or heterospecific competitors that have similar niches. Conspecific examples in birds include collared flycatchers *Ficedula albicollis* increasing their emigration rates from forest patches where the number of nestlings in the area was experimentally lowered [8], bobolink *Dolichonyx oryzivorus* prospectors becoming territorial in habitats with conspecific playbacks, irrespective of habitat quality [9], Northern wheatears *Oenanthe oenanthe* homing in on sites with successful conspecifics in the previous year [10], griffon vultures *Gyps fulvus* locating carcasses based on the flight behaviour of others [11], and great tits copying the specific behaviour of demonstrators in a food accessing puzzle that could be solved in two ways, leading to cultural divergence in this behaviour [12]. Examples of heterospecific attraction mainly comes from literature on great tits and collared and pied flycatchers, where the later arriving flycatchers preferentially copy the local habitat choices of great tits [6, 13–17]. Moreover, flycatcher females avoided patches in which the reproductive timing of great and blue tits had been experimentally delayed [18]. Interestingly, tits have also been suggested to hide social information from flycatchers, as they covered their eggs more when there were flycatcher song playbacks outside their nest box [19]. Heterospecific habitat copying has also been suggested previously in a correlational study, showing that kestrels *Falco tinnunculus* preferentially reoccupied successful roller *Coracias garrulous* nests, and that roller densities increased where kestrels were successful [20]. Social information use has therefore been convincingly shown to cross species boundaries [21]. Relatively few studies have considered conspecific and heterospecific information in the same research. Collared flycatchers not only preferred both nest boxes that were occupied by other flycatchers in previous years, but also boxes that were in the vicinity of Great Tits [22]. Least flycatchers *Empidonax minimus* and American redstarts *Setophaga ruticilla* were attracted to conspecific playbacks, but flycatchers were also attracted to heterospecific playbacks, whereas redstarts showed heterospecific avoidance [23]. Moreover, competitor density cues may play a role in mediating whether conspecific or heterospecific information is preferred. Later arriving collared flycatchers that were faced with a nest box choice conferring either conspecific or heterospecific information preferentially chose the conspecific box in areas dominated by conspecifics, whereas the heterospecific symbol was preferentially chosen in tit dominated areas [7].

Using social information can be costly, because it is generally acquired from competitors. Therefore, there is a theoretical optimum number of competitors, at which the positive effect of the information value outweighs the negative effect of competition [4, 24], which has also been shown experimentally. By manipulating tit densities it was shown that pied flycatchers preferred settling in patches with intermediate densities of tits (quadratic relationship), but the fitness consequences in this study were negatively linearly related to the number of competitors [25]. Moreover, the propensity to use social information can be related to a species’ life history. For example, tits are year-round residents and are generally expected to rely more on personal information than migratory flycatchers with little time between arrival and breeding. In order to gather information or gain nesting opportunities, flycatchers regularly prospect at tit breeding sites [26], which is a potentially deadly strategy as flycatchers are regularly killed by tits in nest cavities [27–29]. Therefore, flycatchers face a trade-off between the cost of competition and the benefits of gaining information about potential breeding sites.

In short, many studies have shown that birds can use both conspecific and heterospecific information (see aforementioned references), but few have attempted to identify which type of information is preferred. It can be argued that conspecifics should be preferred as a source of social information, because these have the same niche. Heterospecifics on the other hand may provide cues that are further advanced in time, and may therefore provide more reliable information about habitat quality [7, 21, 22]. In our study, we closely follow the study design used by Jaakkonen and others [7] to further elucidate how the preference for social cues may depend upon the relative density of conspecific and heterospecific competitors. Although Jaakkonen and others found density dependence of con- or heterospecific information preference, our initial expectation when we started this experiment in 2014 was that conspecific information would be preferred in all contexts.

## Methods

### Study area and species

Our experiments took place in the breeding season (April-June) of 2014 and 2016 in the Netherlands, in the province of Drenthe in Dutch National Park Drents-Friese Wold, subarea Dieverzand (52°52′26″N 6°19′40″E, hereafter “flycatcher dominated”) and Boswachterij Ruinen (52°43′40″N 6°24′00″E, hereafter “tit dominated”), which have a temperate climate [for details study area, see,30]. The experiments were restricted to two study areas, each with 100 nest boxes: Ruinen, an area with more blue and great tits than pied flycatchers, and Diever with more pied flycatchers than tits. Ruinen is dominated by deciduous trees, with pedunculate oak *Quercus robur* being most abundant, and Diever is a more coniferous habitat, dominated by Scots pine *Pinus sylvestris,* intermingled with most small oaks and Silver Birch *Betula pendula*. Nest boxes were spaced by about 40 m in a grid like fashion. The spatial separation between the two areas is about 20 km with very little exchange between populations: six out of 2924 (0.2%) recaptured birds had moved between the two study areas between 2007 and 2016, none of which was during experimental years. In 2016 we added 10 new nest boxes to the tit dominated breeding population, because nest occupation was very high. The nest boxes in the study areas are mostly used by great tits, blue tits, and pied flycatchers, but other species like nuthatch *Sitta europaea* and coal tit *Parus ater* are occasionally found breeding in the study areas too. The great and blue tits are year round residents in the study areas. As a result, tits have the whole year to assess habitat suitability and locate high quality breeding sites. Pied flycatchers on the other hand are long distance migrants that only arrive shortly before the start of breeding, and hence have to decide quickly on potential breeding sites on arrival, and may eavesdrop on the presence and success of resident species. Please be aware that we did this study in two areas over two years, and our results should be interpreted with some caution due to the lack of spatial replication.

### Climate and breeding phenology

The study years in our study plots [30] were characterized by a warm April with early tit phenology in 2014, whereas April 2016 was relatively cold and had a late tit phenology (Table 1). Pied flycatcher arrival and laying dates (1 = 1 April, 31 = 1 May) did not differ strongly between study years (Table 1). Mean male arrival dates in 2014 and 2016 were 17.4 and 15.9 April respectively, and mean female arrival dates were 28.1 and 24.2 April in those years (population average 2007-2016 males 19.1 April, females 26.1 April). Tit densities were slightly above average in 2014, whereas they were high in 2016 (Table 1). In 2014 flycatcher numbers were below average, mostly because a large proportion (>20%) of males remained unpaired [30]. Experiments were performed in years with relatively low caterpillar abundance, and the caterpillar peak in both years was around 20 May.

**Table 1:** Laying dates in April date (1 = 1 April, 31 = 1 May) and number of breeding pairs (in parentheses) of common nest box breeders in all our study plots (1050 nest boxes) in Dwingelderveld, Drents-Friese Wold, and Boswachterij Ruinen [30]. Mean April temperatures are in degrees Centigrade

Year	*F. hypoleuca*	*P. major*	*C. caeruleus*	April T (°C)
2014	35.5 (271)	12.5 (371)	11.9 (109)	11.4
2016	38.7 (308)	24.0 (410)	19.7 (83)	7.9
2007-2016	36.0 (306)	19.4 (355)	18.2 (99)	9.7

### Experimental design

We aimed to investigate how the later arriving pied flycatchers used conspecific or heterospecific cues when selecting a nest box. In our experiment we first relied on the natural occupation of nest boxes by tits (early in the season), and the settlement of the first arriving 50% of the pied flycatchers, which started from the second week of April. Pied flycatcher arrival, a repeatable trait in our population, was monitored once every two days, both for males and females [31]. The species occupying a nest box was determined by the singing of a pied flycatcher male near a nest box and/or nest building inside a nest box. The latter was possible because pied flycatchers and tits use different nest materials. Nests of tits mostly consist of mosses with feathers and hairs [32, 33] and pied flycatchers build their nests with dead leaves, pieces of bark (pine or birch) woven with grasses [34, 35].

### Flycatcher arrival

**Fig. 1:**
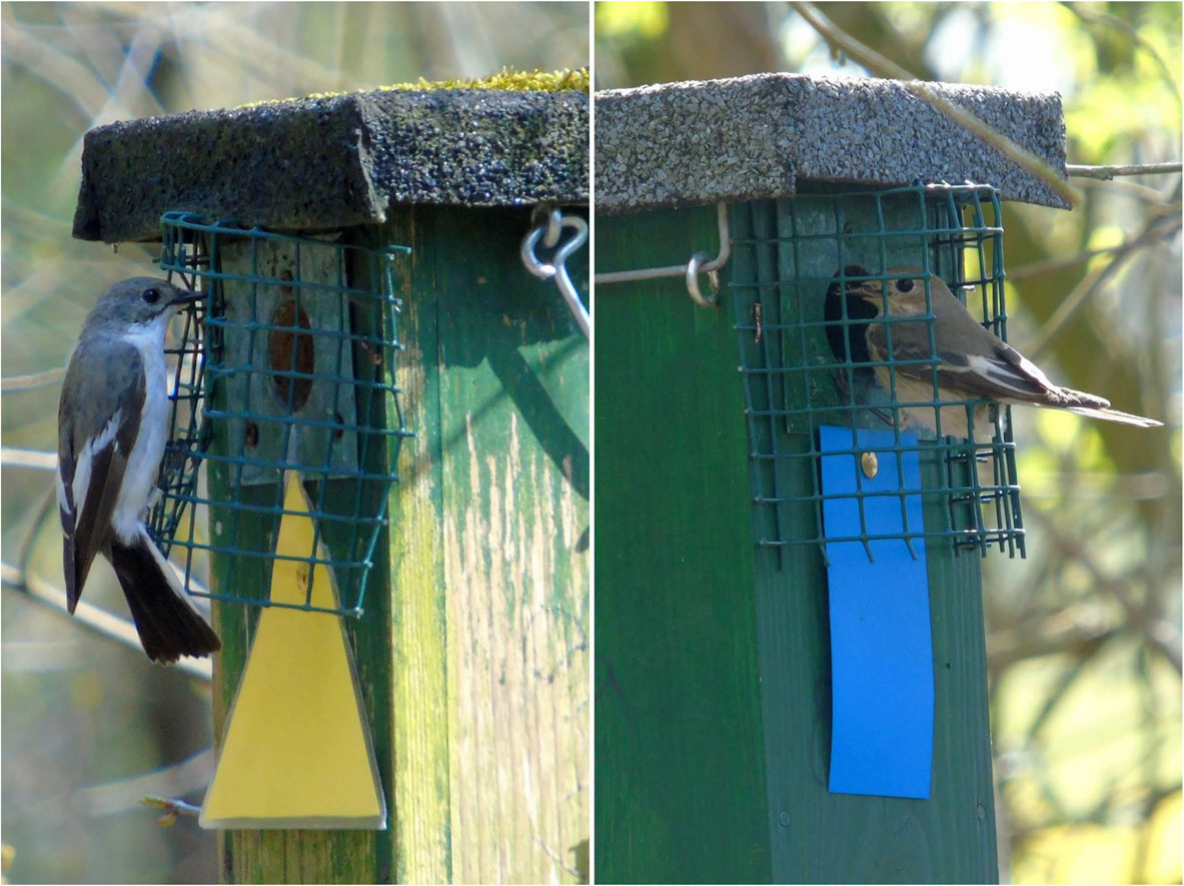
A male Pied Flycatcher at a nest box with a yellow triangle symbol (left panel), and a female at a nest box with a blue rectangle symbol (right panel)

**Fig. 2:**
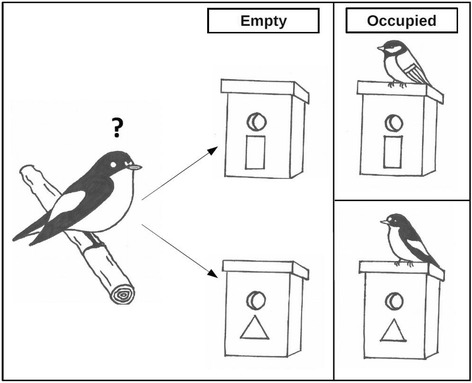
Experimental setup. Later arriving male Pied Flycatchers (left) could choose between empty nest boxes with a blue rectangle or yellow triangle attached to it (middle). Within a study area, these symbols represented either the manipulated nest site character preference of heterospecific tits (top right) or conspecific early arriving flycatchers (bottom right). The symbol distribution was swapped between areas and years

When approximately 50% of the expected flycatcher males had arrived in the study plots (based on previous year’s numbers), the experiment was initiated. Each nest box received an artificial symbol that was clearly distinguishable. Such artificial geometric symbols have been previously successfully implemented to study social information use in cavity nesting passerines [6, 7, 13–16, 36, 37]. Two different symbol types were used: a yellow triangle and a blue rectangle (Fig. 1). All nest boxes in an area that were occupied by tits received a certain symbol (for example the yellow triangle; further referred to as the heterospecific symbol) and all nest boxes in the area that were occupied by the expected first 50% of male pied flycatchers received the other symbol (in this example the blue rectangle, called the conspecific symbol). Next we randomly allocated half of the empty nest boxes in the area with the heterospecific symbol, and the other half with the conspecific symbol. By doing this, nest site information was manipulated: it appeared to newly arriving pied flycatchers that tits had settled in one type of box and flycatchers in the other box type (Fig. 2). Pied flycatcher males that arrived after initiation of the experiment had to choose between nest boxes that had either a conspecific or a heterospecific signal (triangle or rectangle). Since there was a possibility that birds have a preference for a symbol due to its color or shape, we decoupled the association between conspecific and heterospecific information by swapping symbol types between years and areas.

### Determining symbol choice

The choice between a conspecific and a heterospecific nest box was determined by the presence of a pied flycatcher male singing at the nest box or nest building activity taking place inside the nest box. Every 2-3 days, all empty nest boxes and tit nest boxes were checked for flycatcher settlement. Every two to seven days (average = 2.3 days), the status of all nest boxes was determined during a full plot check. We slightly changed the experimental setup between 2014 and 2016. In 2014 the symbols were left as they were implemented on the day the experiment started, whereas in 2016 we gave newly arrived flycatchers a flycatcher symbol when they had settled at a nest box with tit symbol. This meant that flycatchers always had a flycatcher symbol in 2016, whereas some had a tit symbol in 2014 (7.4% in the tit dominated area, 4.3% in the flycatcher dominated area in the middle of the experiment). This may have slightly diluted information reliability in 2014, but since the vast majority of flycatchers still had a flycatcher symbol, we believe this had a marginal effect on the choices made. Moreover, in the year 2014 the experimental design was implemented too late in the tit dominated area, which resulted in a small sample size (flycatcher choices) of *n* = 7. The experiment was not performed in 2015, which resulted in only three flycatchers being present in both experimental years. Individual preference therefore could hardly have affected the results presented here.

### Data processing

Data was ordered at the nest box level, and flycatcher choice was assigned binomially depending on whether a box had been occupied by a flycatcher or remained empty. In some cases tits had abandoned their nest box and so these became available to flycatchers. Such choices were treated as normal choices, because we could not discern between nest abandonment and take-overs. We did however assign nest material presence or absence binomially, and used this as a covariate for later analyses, since it is known that flycatchers prefer boxes with nesting material present [38]. For each of the available nest boxes it was known what symbol it received when the experiment started and whether it was eventually chosen by a flycatcher (Table 2). In total, 154 nest boxes were available over the two years and 73 of these were finally chosen by a pied flycatcher male. 38 of the 82 heterospecific nest boxes and 35 of the 72 conspecific nest boxes were chosen by pied flycatcher males (Table 3).

**Table 2:** Overview of the number of tits and flycatchers per area at the start and end of the experiment in two nest box populations shows differences in relative abundance of tits and flycatchers at the start and end of the experiment. Ruinen had more heterospecific (tits) than conspecific (flycatchers) tutors, whereas the opposite was true for Diever

	Ruinen 2014	Diever 2014	Ruinen 2016	Diever 2016
Tits/flycatchers start #	56 / 27	35 / 31	60 / 13	29 / 26
Tits/flycatchers end #	56 / 34	35 / 49	60 / 38	29 / 49
Flycatchers/all birds start %	32.5%	47.0%	17.8%	47.3%
Flycatchers/all birds end %	37.8%	58.3%	38.8%	62.8%

**Table 3:** Frequency of available and chosen nest boxes (chosen boxes/available boxes) by Pied Flycatchers in an experiment providing conspecific and heterospecific symbols on nest boxes. The experiment was conducted in a tit dominated (tit rich) and a Pied Flycatcher dominated (PF rich) area (Table 1)

	2014			2016		
Area	*Tit symbol*	*PF symbol*	*Total*	*Tit symbol*	*PF symbol*	*Total*
Tit rich	5/12	2/11	7/23	18/29	7/20	25/49
PF rich	4/11	14/16	18/27	11/30	12/25	23/55
Total	9/23	16/27	25/50	29/59	19/45	48/104

### Statistical analysis

Statistical analysis was done using R version 3.2.4 Revised [39]. The “glmer” function from the R package “lme4” [40] was used to fit a binomial generalized linear mixed-effects model (GLMM). The nest box choice (binomial, chosen or not chosen) was the response variable. Fixed predictor variables contained “Area” (tit or flycatcher dominated), “Information type” (heterospecific or conspecific symbol), and the interaction between them, as we expected the choice may have been modified by majority information. We also added the presence of “nest material” before the flycatcher choice as a fixed effect. Since data from 2014 and 2016 were combined, “Year” was added as a random intercept. In the results, be aware that the fractions of nest box types “chosen divided by available” do not need to add up to one (they can add up to 2 if all boxes had been occupied), as these data are not only comprised of the choices of flycatchers, but also of the unchosen boxes. Our setup required us to analyze the data at the nest box level, because unlike in a paired nest box setup [15], flycatcher choices in our experiment were not independent of availability. Therefore, an analysis that only considers the chosen and not the unchosen nest boxes could over- or underestimate the effect.

## Results

**Fig. 3:**
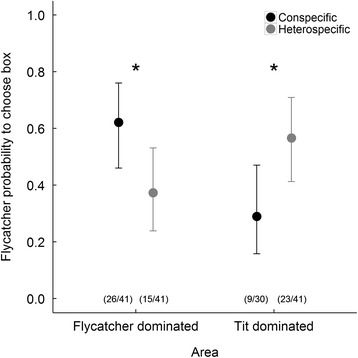
Probability that a nest box with a heterospecific (in blue) or conspecific (in red) characteristic in a flycatcher rich (left) or tit rich (right) area is chosen by a male Pied Flycatcher in an experiment providing conspecific and heterospecific symbols on nest boxes. Whiskers indicate 95% Confidence Interval (Table 3). Be aware that data points are independent of each other, and do not necessarily add up to 1. Sample sizes stated in parentheses

Pied flycatchers were more likely to choose a nest box with a conspecific symbol in the Pied flycatcher dominated area (*p* = 0.033) and more likely to choose a nest box with a heterospecific symbol in the tit dominated area (Fig. 3, Table 4, *p* = 0.022), showing that pied flycatchers copy whoever forms the majority, whether they are conspecific or heterospecific competitors. Moreover, the choice ratios between the two areas differed significantly from each other (interaction area*information type: *p* = 0.0016, Table 4).

**Table 4:** The nest box choice of later arriving male Pied Flycatchers in tit and flycatcher dominated areas, modeled as the probability that an available nest box with a certain nest site character was chosen (baseline model conspecific choice, flycatcher dominated). Conspecific information was copied preferentially in flycatcher dominated areas, whereas the opposite was true in tit dominated areas (Fig. 3)

Box chosen (1/0)^a^	Estimate (SE)	Z_5,149_	*p*-value
Intercept (flycatcher dominated, conspecific)	0.580 (0.328)	1.770	0.077
Area tit dominated	−1.391 (0.518)	−2.687	0.0072
Information type heterospecific	−1.014 (0.476)	−2.129	0.033
Nest material presence	−0.242 (0.370)	−0.656	0.512
Area^a^Information type	2.177 (0.689)	3.161	0.0016

^a^Random effect variance ± SD ‘1 | year’ = 0.000 ± 0.000

## Discussion

We found that pied flycatchers use social information when selecting a nest site, but that the preference for conspecific or heterospecific information depended on the density of either tits or flycatchers. Assuming that both heterospecific and conspecific cues are reliable, we expected to find a preference for conspecific information, because we expected intraspecific information to overrule interspecific information. Our results suggest that the preference for a nest box with either the heterospecific or conspecific symbol depended on the abundance of heterospecific and conspecific cues in an area. More specifically, we found that pied flycatchers had a preference for the information type that was in the majority, regardless of which species conveys this majority information. When initiating our experiment in 2014, we were unaware that the same experiment had been performed in a Swedish population of tits and collared flycatchers [7]. The striking similarity between both studies demonstrates that the flexible incorporation of both conspecific and heterospecific social information is a persistent mechanism in habitat selecting flycatchers.

Our experiment was based on settlement decisions of late (last ~50%) pied flycatchers, and our findings are in accordance with earlier studies where later arriving, relatively inexperienced individuals have a propensity to use social information [6, 7]. In our population, later arriving individuals are on average younger individuals [31], which is in line with expectations that it is more beneficial to eavesdrop on others when you are relatively inexperienced or uncertain [5]. Moreover, the result that pied flycatchers use information of the species that is in the majority is in accordance with a previous study where in late spring, collared flycatchers preferred a nest box with a tit symbol when the number of tit tutors was high and the flycatcher symbol when the number of tit tutors was low [7].

Our study showed that pied flycatchers are able to use arbitrary symbols as an information cue. This is to some extent remarkable, because geometric symbols are not generally encountered in natural situations. However, together with previous studies using similar symbols, our findings support the use of arbitrary symbols as a successful method to study social information use in birds [6, 7, 13–16, 36, 37]. But why do pied flycatchers respond so strongly to geometric symbols at all? In natural situations, characteristics of a chosen nest site reflect the preference of the tutor, giving information about the value of that nest site itself, but in our case the nest site characteristic was completely artificial. Nevertheless, using artificial setups to study questions about behavioural copying is not uncommon. For example, animals from chimpanzees *Pan troglodytes* to great tits were able to learn different strategies in how to get food from a human introduced apparatus [12, 41]. Animals are apparently quite flexible in being able to incorporate new situations in their behavioural decisions. It can even be argued that using experimental setups that animals would never encounter in nature allows researchers to eliminate the possibility of innate or personally learned preference, so that we can draw strong inference about social learning as the sole mechanism explaining such patterns.

When heterospecific and conspecific information are both useful, cue frequency apparently explains why a preference for either is found in our study. Frequency dependent cue using or majority copying has been found in quite a few studies, but has seldom been shown to cross species boundaries. For example, the number of demonstrators enhanced following behaviour of naïve guppies *Poecilia reticulata* [42], rock doves *Columba livia* learned how to open an inverted test tube quicker when the number of demonstrators was higher [43], Norway rats *Rattus norvegicus* ate previously perceived unpalatable food when demonstrator rats ate it [44], and nine-spined sticklebacks *Pungitius pungitius* were more likely to follow the foraging behaviour of larger groups of demonstrators [45]. Jaakkonen and others [7] showed that collared flycatchers may also copy heterospecific majority information, but to our knowledge there are no other studies on this topic. Although it had been found that novel preferences can be obtained from heterospecifics [6], most studies did not weigh this in relation to conspecific information.

## Conclusions

Our experiment independently shows that information of heterospecific individuals can be preferred in the presence of conspecific minority information. The integration of both conspecifics and heterospecifics in this study is a more realistic reflection of the ecological fluctuations that animals face in choosing a breeding site. It would be interesting to focus future experimental research on whether behavioural copying is transmitted faster between conspecifics or heterospecifics, and determining threshold values of conspecific versus heterospecific information preference.
